# The role of ferroptosis in liver injury after cold ischemia–reperfusion in rats with autologous orthotopic liver transplantation

**DOI:** 10.1007/s10047-024-01488-2

**Published:** 2025-01-06

**Authors:** Wei Wu, Bei Xu, Haibin Huang, Ying Mao, Yuan Gao, Wenhao Bu

**Affiliations:** 1 Department of Anesthesiology, CR & WISCO General Hospital, Affiliated to Wuhan University of Science and Technology, Wuhan, 430080 China; 2 Department of Anesthesiology, Maternal and Child Health Hospital of Hubei Province, Tongji Medical College, Huazhong University of Science and Technology, Wuhan, 430070 China

**Keywords:** Liver transplantation, Liver injury, Ferroptosis, Ferrostain-1, GPX4

## Abstract

Using autologous orthotopic liver transplantation (AOLT) model in rats, the effect of lipid reactive oxygen species (L-ROS) inhibitor Ferrostain-1 on ferroptosis signal pathway was observed to determine whether ferroptosis occurred in rat liver injury after cold ischemia–reperfusion (I/R). Thirty-two healthy adult SPF male SD rats, 8 ~ 10 weeks old, weight 240 ~ 260 g, were divided into four groups by the method of random number table (*n* = 8): sham group, I/R group, I/R + Fer-1 group, I/R + DFO group. In the I/R + Fer-1 group, ferristatin-1(5 mg /kg) was intraperitoneally injected 30 min before surgery; in the I/R + DFO group, DFO 100 mg/kg was injected intraperitoneally 1 h before operation and 12 h after operation. Blood samples were taken from the inferior hepatic vena cava 24 h after reperfusion. After anesthesia, the rats were killed and part of their liver tissue was removed. The pathological changes of liver tissue sections were observed under a high-power microscope, and the liver injury was evaluated. Serum malondialdehyde (MDA) and serum levels of ALT, AST and IL-6 were determined by the ELISA method, Reduced glutathione (GSH), glutathione peroxidase 4 (GPX4), MDA, Fe2 + and superoxide dismutase (SOD) were determined in the liver tissue. Compared with the sham group, the serum levels of the IL-6,MDA, AST and ALT in I/R group were obviously higher (*P* < 0.05); The levels of MDA and Fe^2+^ in liver tissue were significantly increased (*P* < 0.05).The levels of SOD, GSH and GPX4 in liver tissue were decreased. The levels of serum MDA, IL-6, AST, and ALT in the I/R + Fer-1 and I/R + DFO groups were significantly lower than those in the I/R group at 24 h after reperfusion. In the I/R + Fer-1 group, the level of MDA in liver tissue decreased significantly, while the level of SOD, GSH and GPX4 in intestinal tissue increased (*P* < 0.05). In The I/R + DFO group, the levels of MDA and Fe^2+^ in liver tissue decreased significantly, while the level of SOD in intestinal tissue increased (*P* < 0.05). Ferroptosis is involved in pathophysiological process of liver injury after cold ischemia–reperfusion in AOLT rats.

## Introduction

Liver ischemia–reperfusion (I/R) injury is a major problem during liver transplantation (LT). Perioperative LT can not only lead to liver injury, but also induce severe systemic inflammatory reaction and distant organ injury [1–3]. Ferroptosis is a new programmed cell death with special iron dependence that is characterized by the accumulation of intracellular lipid reactive oxygen species (L-ROS), which involves inhibition of glutathione biosynthesis or small molecules of glutathione peroxidase 4 (GPX4) (a glutathione-dependent antioxidant enzyme), resulting in mitochondrial damage [4].When ferroptosis occurs, cells trigger the inherent immune system and release damaging molecules related to inflammation, which causes immune cells to stimulate the body to produce inflammatory response by recognizing different modes of cell death [5]. Some studies have shown that ferroptosis is related to perioperative intestinal and liver injury during LT [6, 7]. However, whether the liver injury after cold ischemia–reperfusion in the perioperative period of LT is related to ferroptosis remains to be discussed. This study is based on the rat AOLT model to investigate the role and possible mechanism of ferroptosis in liver injury after LT in rats.

## Methods and materials

### Experimental animals and group details

The experimental scheme and implementation procedure have been approved by the Experimental Animal Ethics Committee of Guilin Medical University (GLMC-IACUC-2021014). Thirty-two healthy adult SPF male SD rats, 8–10 weeks old and weighing 240-260 g, were purchased from Hunan Shrek Jingda Experimental Animal Co., Ltd. (SCXK (Hunan) 2019–0004). The rats were raised in the Animal Experimental Center of Guilin Medical University at room temperature 22–25 ℃, relative humidity 35–60%, alternate light:dark cycle for 12 h and free diet. After a week of adaptive feeding, rats were divided into four groups by the method of random number table (*n* = 8): sham operation group (sham group), AOLT group (I/R group), AOLT + ferroptosis inhibitor ferrostain-1 group (I/R + Fer-1 group), AOLT + iron chelator deferoxamine (DFO) group (I/R + DFO group). In the sham group, only laparotomy was performed and the abdomen was closed after the corresponding blood vessels were dissociated. The AOLT model was established in the other three groups. In the I/R + Fer-1 group, 5 mg/kg of ferrostain-1 (batch number: HY-100579,MCE Company) was injected intraperitoneally 30 min before operation and dissolved with DMSO 2 ml. In the I/R + DFO group, DFO (batch number: HY-B0988,MCE) 100 mg/kg was injected intraperitoneally 1 h before operation and 12 h after operation.

The rats were euthanized before the liver samples were taken out. Necessary measures were used to minimize the animals’ suffering during the experiment.

### Model establishment

**Fig. 1:**
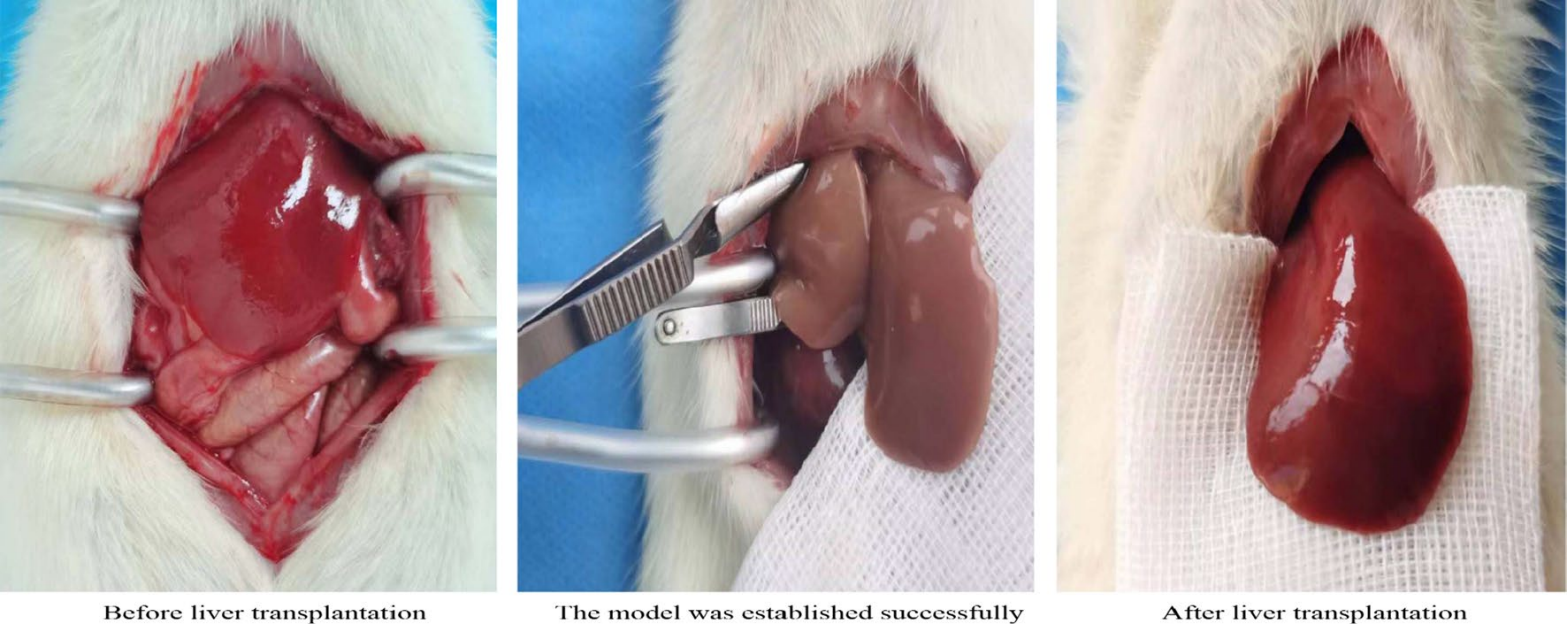
Color changes of rat liver in three different periods

For establishment of the AOLT model [8], The rats were fasted for 12 h before surgery and drank water freely, until 2 h before surgery. Rats were injected intraperitoneally with 3% sodium pentobarbital (0.2 ml/100 g) for anesthesia maintenance before surgery, and 0.005% fentanyl (0.16 ml/100 g) was injected intraperitoneally to relieve pain, After the supine position was taken and fixed, the femoral artery was punctured under skin incision, and the transducer was connected to continuously monitor the arterial blood pressure and observe the respiration of the rats. The abdomen depilates and prepares the skin, carries on the disinfection with the iodophor, spreads the aseptic hole towel, cuts along the abdominal midline. After laparotomy, the left triangular ligament and hepatic falciform ligament were dissociated, and the inferior hepatic vena cava (IHVC) and superior hepatic vena cava (SHVC) were carefully and bluntly separated, so that they were fully exposed. The hepatoduodenal ligament was cut open to separate the proper hepatic artery and hepatic portal vein (PV). IHVC, PV and proper hepatic artery were blocked with a non-invasive hemostatic clamp. Above the blocking site of PV, 1 ml of saline containing 30U heparin was infused after puncturing the scalp with a needle. The blood in the liver was allowed to enter the systemic circulation through SHVC, then the SHVC was blocked with a hemostatic clamp. After starting the anhepatic phase and time, normal saline was continuously infused at 4 ℃ through the PV. A small opening was cut above the blocking site of the IHVC as an outflow channel, and the fluid circulating in the liver discharged at a perfusion time of 30 ± 1 min. After perfusion, the liver gradually changed from dark red to khaki. After perfusion, the outflow tract in IHVC and the puncture hole in PV were sutured with 8–0 non-invasive vascular suture, The proper hepatic artery, PV, IHVC and SHVC were opened successively, and the vascular reflow was made to restore the liver color to bright red. After the operation, the abdominal cavity was rinsed with 36℃ normal saline and carefully sutured layer by layer (Fig. 1).

### Specimen collection

24 h after the end of ALOT in the rat, the rat was anesthetized again and the abdominal cavity opened. 2 ml of inferior vena cava blood was taken and placed in a BD tube. A high-speed centrifuge was used to prepare serum samples, and which were stored in a refrigerator at-80℃ for later use. After successful collection of blood from the inferior vena cava, 50 ml of heparinized physiological saline at 4 °C was slowly injected into the left ventricle of the rat, and a small opening was cut in the right atrial appendage to serve as the outflow tract. By fully perfusing heparinized physiological saline at 4 °C, residual blood in each organ was washed as thoroughly as possible, and then the liver tissue of the rat was taken out and stored in a refrigerator at-80° C for later use.

### Details of testing indicators

#### The pathological changes of the liver of rats in each group were observed under high magnification

Partial rat liver tissue was fixed with 4% paraformaldehyde solution for over 24 h. After embedding the liver tissue specimen in paraffin, the liver tissue specimen was sectioned (with a thickness of 5 μm) using a microtome and gradually stained with H&E. The pathological changes of liver tissue sections were observed under a high-power light microscope (EVOS M5000, Thermo Fisher Scientific, USA) (× 200). The operation was carried out by two pathology professionals from the Department of Pathology of the Affiliated Hospital of Guilin Medical University.

#### Detection of serum ALT and AST levels in rats

The serum was thawed and the levels of AST and ALT in serum of mice were detected by the AST and ALT detection kits, respectively, according to the instructions of the kit.

#### Determination of serum IL-6 levels in rats

IL-6 ELISA KIT (item number: SEKR-0005, Beijing Solebo Biotechnology Co., Ltd.) was used to detect the level of inflammatory cytokines (serum IL-6) in rat serum.

#### Analysis of MDA content in liver tissue and serum

He maximum absorption peak was at 532 nm, and the peroxide product MDA combined with TBA to form a red product. An instrument was used to measure the OD value of the sample, and then the MDA content calculated. The degree of lipid oxidation was assessed by determining the MDA concentration. In short, 0.1 g of rat liver tissue was collected, and the homogenate was prepared and centrifuged at 8000 g at 4 °C for 10 min. The supernatant was collected and placed on crushed ice for testing. After centrifuging the anticoagulated blood, the upper serum was collected, and the MDA content in liver tissue and serum was measured using a lipid diamine peroxide content detection kit (catalog no.: BC0025, Beijing Solarbio Science & Technology Co., Ltd.).

#### Determination of SOD activity

Superoxide dismutase (SOD) is the main enzyme of antioxidation in the body, which can protect the cells from oxidative damage by scavenging ·O2^–^. The instructions of the SOD activity test kit (item number: BC0170, Beijing Solebao Biotechnology Co., Ltd.) was strictly followed for testing.

#### *Determination of Fe*^*2*+^*content in liver tissue*

0.1 g of liver tissue and 1 ml of extract were mixed and homogenized in an ice bath, centrifuged at 4000 g for 10 min at 4 °C and the supernatant collected. The Fe^2+^ content in the supernatant of liver tissue samples was detected using a tissue iron content detection kit (Beijing Solarbio Science & Technology Co., Ltd., catalog number: BC4355).

#### GPX4 activity in liver tissue

0.1 g of rat liver tissue stored at -80° C was completely ground using a fully automatic rapid sample grinder, and 1 ml of extract was added to make tissue homogenate. After centrifugation, the supernatant was collected and stored at low temperature for later use. The instructions of the GPX4 detection kit (catalog no.: K003083P Beijing Solarbio Science & Technology Co., Ltd.) were strictly followed to detect the GPX4 content in liver tissue. Then, the OD value of the sample was obtained at a wavelength of 450 nm, and the results were analyzed to calculate the GPX4 content.

#### The GSH content in liver tissue

1. 0.1 g of frozen liver tissue was weighed and fully ground using an automatic sample rapid grinder (Shanghai Jingxin, China), and 1 ml of extract was added to prepare liver tissue homogenate. The samples were then centrifuged at 8000 g for 10 min at 4℃; the supernatant was taken and stored at 4 °C for subsequent analysis.
2. Anticoagulated blood was centrifuged at 600 g at 4 °C for 10 min. The upper plasma was transferred to another PE tube and centrifuged at 8000 g for 10 min at 4 °C. The supernatant was transferred to a new tube and stored at 4 °C. GSH content in liver tissue and serum was detected using reduced GSH content detection kit (catalog no.: BC1175, Beijing Solarbio Science & Technology Co., Ltd.).

### Statistical analysis

A database was established and SPSS26.0 used for statistical analysis. Data conforming a normal distribution are expressed as mean and standard deviation(‾*x* ± *s*). Repeated measures analysis of variance was used to compare differences between groups, and then t tests were used to compare differences between the two groups. *P* < 0.05 was considered statistically significant.

## Results

### ***Histopathological changes of liver tissue (***Fig. 2***)***

**Fig. 2:**
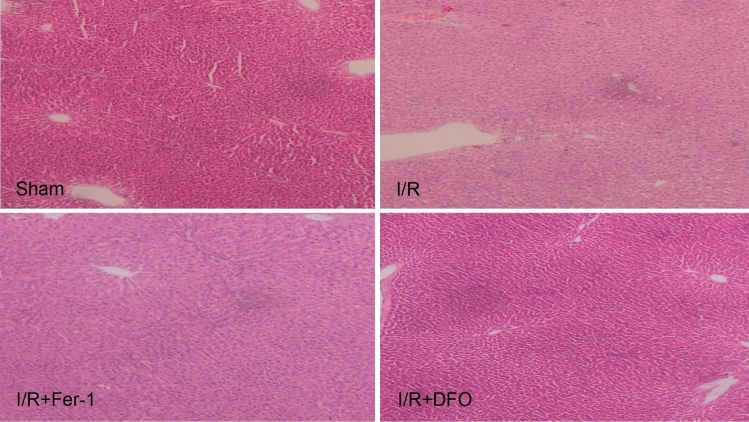
Comparison of the pathological changes of liver tissue by HE staining between groups (× 200)

The results of HE staining showed that the liver tissue of the sham group was clear, the structure was complete, the cells were arranged neatly and there was no inflammatory cell infiltration. On the other hand, large-scale histiocyte necrosis, infiltration of inflammatory cells, loose arrangement of cells and reticular distribution were found in the I/R group. Compared with the I/R group, the pathological condition of the I/R + Fer-1 group liver tissue was significantly improved, the structure and nucleus of hepatic lobules gradually became clear, the inflammatory infiltration gradually decreased and the arrangement of cells gradually became regular. In the I/R + DFO group, the uniform size of hepatocytes tended to the normal level, the intercellular space and cell outline became more regular and clear and the inflammatory infiltration of cells was significantly improved.

### ***Serum MDA, IL-6, AST and ALT levels (***Fig. 3***)***

**Fig. 3:**
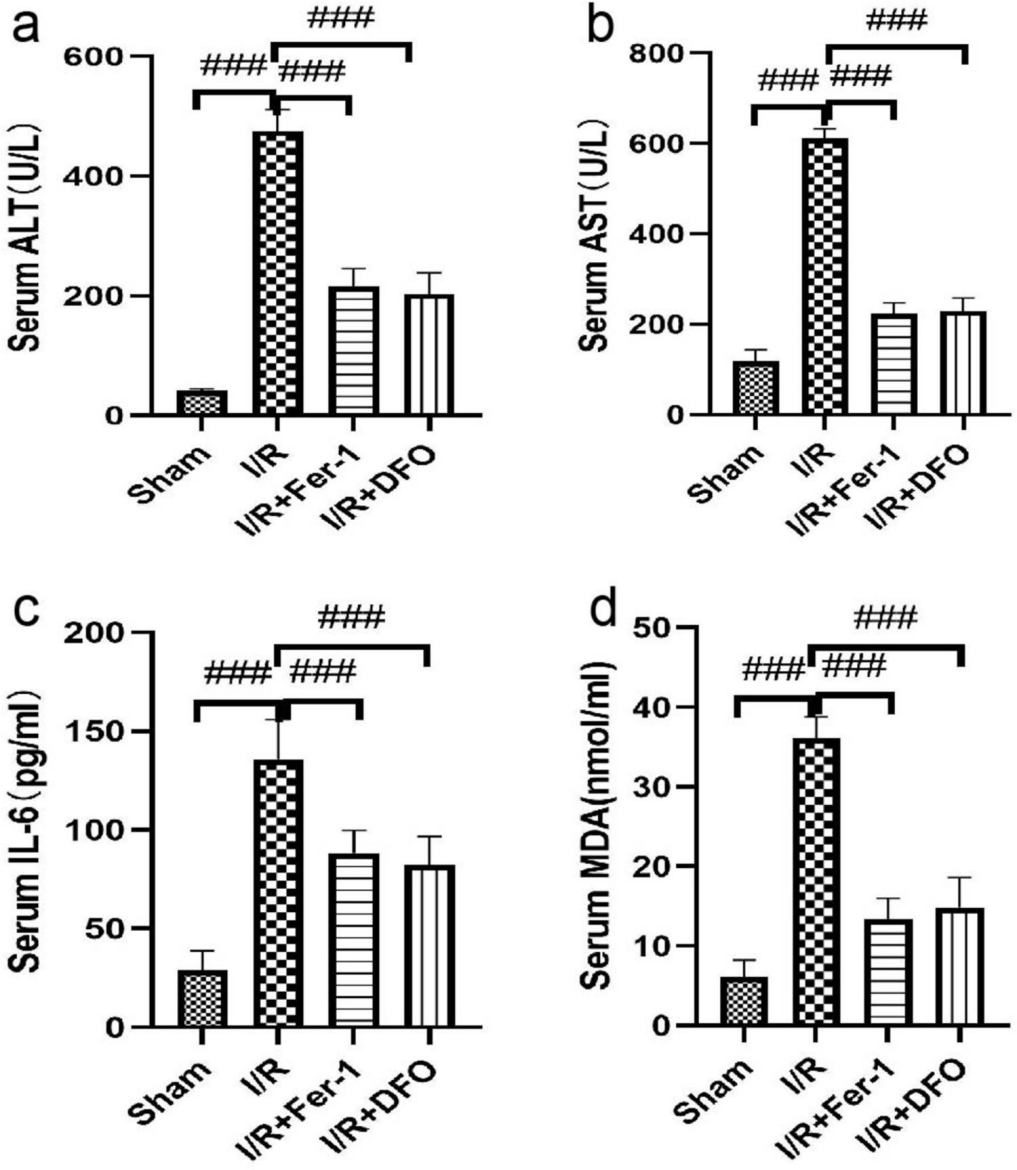
(a,b,c,d) Changes in ALT, AST, IL-6 and MDA in serum 24 h after the anhepatic stage. The results are expressed as the mean ± SD (*n* = 8 per group). *n* = 8. * *p* < .05, ** *p* < .01, *** *p* < .001,****p < .0001 by t tests

Compared with the sham group, the serum IL-6, MDA, AST and ALT contents in the other three groups were significantly increased (*P* < 0.05). The levels of MDA, IL-6, AST and ALT in the serum of rats in the I/R + Fer-1 and I/R + DFO groups were lower than those in the I/R group 24 h after reperfusion (*P* < 0.05). Compared with the I/R + Fer-1 group, there was no significant difference in serum indexes between the I/R + DFO group and I/R + DFO group.

### ***Liver tissue MDA, SOD and liver Fe***.^***2***+^***, GSH,GPX4 levels (***Fig. 4***)***

**Fig. 4:**
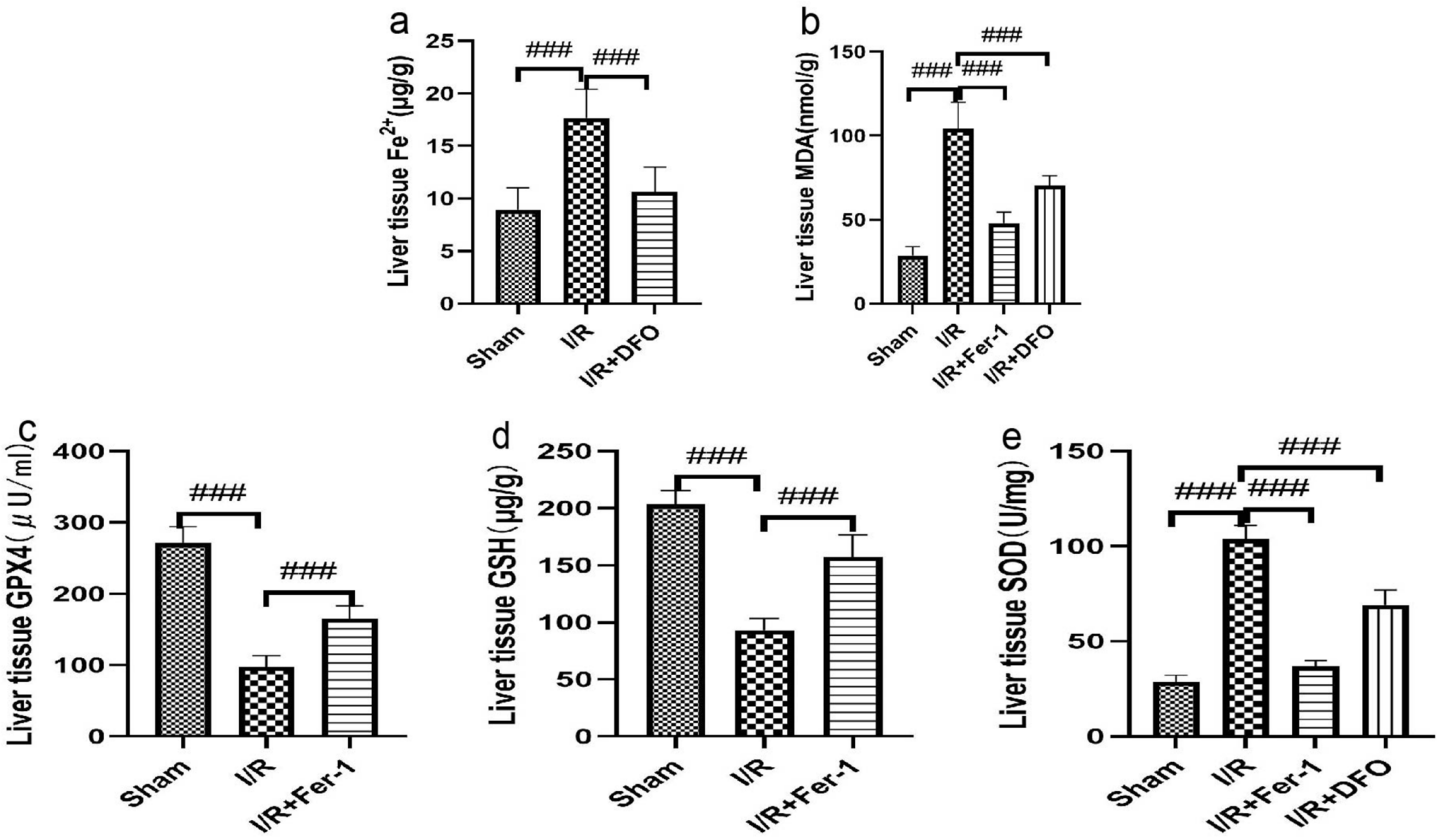
(a,b,c,d,e) The levels of Fe2 + , SOD, MDA, GSH and GPX4 in liver tissue of rats in each group were measured 24 h after the anhepatic period. The data are shown as the means ± S.D (*n* = 8). * *p* < .05, ** *p* < .01, *** *p* < .001,****p < .0001 by t tests

Compared with the sham group, the levels of MDA and Fe^2+^ in liver tissue of the other three groups were significantly higher, while the levels of SOD, GSH and GPX4 in liver tissue were lower. (P < 0.05). Compared with the I/R group, the level of MDA in the liver tissue of the first group decreased significantly 24 h after reperfusion (P < 0.05). Elevated levels of SOD, GSH and GPX4 were found in the intestinal tissue (P < 0.05). The levels of MDA and Fe2 + in liver tissue decreased significantly in the I/R + DFO group (P < 0.05). There was elevated level of SOD in the intestinal tissue (P < 0.05).

## Discussion

This study refers to the literature [8] to establish the model of AOLT in rats. The results showed that compared with the sham operation group, liver tissue cells in the I/R group showed necrosis, infiltration of inflammatory cells and loose cell arrangement, suggesting that the model of liver injury in rats with AOLT was established successfully. Ferrostain-1 is an effective and selective ferroptosis inhibitor, which can increase the expression of GPX4 and reduce lipid peroxidation. It can avoid membrane lipid damage and inhibit cell ferroptosis through a reduction mechanism [9]. The chelating agent deferriamine (DFO) can convert free iron ions into stable compounds, preventing iron from supplying electrons to oxygen to form reactive oxygen species, and recent studies have shown that DFO is also an ferroptosis inhibitor [10].This study was treated by intraperitoneal injection of ferrostain-1 and DFO according to the pre-experiment.

Ferroptosis plays an important role in many pathophysiological processes such as ischemia–reperfusion injury of various organs [11]. It is characterized by iron overload and membrane lipid peroxidation. The process of ferroptosis is accompanied by a large amount of accumulation of intracellular Fe^2+^. Excessive Fe^2+^ induces cell death by promoting lipid peroxidation through Fenton reaction. System Xc^−^ is one of the key pathways of ferroptosis, and GSH, GPX4 and SLC7A11 are important proteins and markers of the System Xc^−^ signaling pathway. When the cystine transport protein is inhibited, the intracellular GSH is exhausted, which eventually leads to the inactivation of GPX4, leading to the accumulation of lipid peroxidation and cell death to a certain extent. The direct inhibition of GPX4 enzyme can also lead to this effect [12–16]. SLC7A11 specifically transports cystine into cells, converts it into Cys under the action of reductase and reacts with Gly and Glu to synthesize GSH, in which Cys can limit the efficiency of GSH synthesis [17, 18].GSH is an important antioxidant in the body. GSH and GPX4 work together to reduce lipid peroxides to non-toxic alcohols and maintain a stable state of redox [19, 20]. SLC7A11 is also a key protein in the negative regulation of ferroptosis. Inhibition of SLC7A11 can induce ferroptosis. Ferritin is a highly stable and widely expressed protein, which is composed of ferritin light chain (FTL) and FTH1 polypeptide chain. FTH1 catalyzes the oxidation of Fe^2+^, while FTL plays an important role in the storage of Fe^3+^. These two chains play a vital role in maintaining the dynamic balance of iron and preventing iron overload [21–25]. FTH1 is a key subunit of ferritin and participates in a variety of disease signaling pathways.

Inflammation plays an important role in the process of liver I/R injury. In fact, we observed that I/R upregulated the expression of inflammatory cytokines in the liver, which was blocked by Fer-1, suggesting that ferroptosis-mediated cell death is an upstream event of inflammatory response in the process of liver I/R injury. In recent years, inflammation caused by necrotizing cell death is called necrotizing inflammation [26–28].In particular, necrotizing inflammation caused by ferroptosis has received considerable attention.

As a kind of pathological cell death, ferroptosis is related to liver I/R. Iron overload is a risk factor of liver I/R in mouse liver I/R model, Iron chelating agents or lipid peroxidation scavengers can reduce liver injury. Therefore, the inhibition of ferroptosis may be a potential therapeutic strategy for the treatment of liver I/R. However, the underlying mechanism of ferroptosis in I/R during the perioperative period of LT is not fully understood [29–31]. New evidence suggests that lipid metabolism is the basis for the formation of ferroptosis, and reshaping lipids may alleviate ferroptosis and liver I/R [32, 33].

The results of this study show thatcompared with the sham group, the content of MDA increased, the content of GSH and GPX4 decreased and the content of Fe^2+^ increased in the I/R group, suggesting that ferroptosis was involved in liver injury during the perioperative period of LT in rats. Compared with the I/R group, the content of MDA in the I/R + Fer-1 group decreased, while the content of GSH and GPX4 increased, and the content of Fe^2+^ decreased in the I/R + DFO group, suggesting that the liver injury of AOLT was alleviated after inhibition of ferroptosis. These results suggest that iron overload is a new risk factor for I/R injury in LT, and ferroptosis is involved in the pathophysiological process of liver injury after cold ischemia–reperfusion after AOLT in rats.

## Conclusion

To sum up, it is suggested that ferroptosis is involved in the pathophysiological process of cold ischemia–reperfusion liver injury after AOLT in rats.

## Data Availability

The datasets used and/or analyzed during the current study are available from the corresponding author on reasonable request.
